# FAST-PB: An automated plant bioengineering system for scalable genome editing and phenotyping

**DOI:** 10.1093/plcell/koaf020

**Published:** 2025-01-31

**Authors:** Nitin Uttam Kamble

**Affiliations:** Assistant Features Editor, The Plant Cell, American Society of Plant Biologists; Indian Institute of Science Education and Research, Thiruvananthapuram 695551, India

Plants, with their innate ability to perform photosynthesis, serve as major sources of food and feed. Recent developments have also enabled plants to be transformed into biofactories capable of producing economically important molecules and therapeutic agents. With the increasing global population, changing climatic conditions, and the increasing probability of more frequent pandemics, the need for better therapeutic agents and enhanced food, fuel, and fiber production is increasing. To meet the demand of economically important molecules, there is a need to scale up production, a goal that can be achieved through plant bioengineering. Plant bioengineering involves several complex steps, including the design of constructs for the gene of interest, plant transfection or transformation, gene mutations, or genome editing, as well as screening and analysis for the desired traits. These are often low-throughput, time-consuming, and labor-intensive processes (Huang et al. 2022).

In this issue of *The Plant Cell*, **Jia Dong, Seth W. Croslow, and colleagues** (Dong et al. 2025) introduced a fast, automated, scalable, and high-throughput pipeline for plant bioengineering, called FAST-PB (Figure). This automated biofoundry integrates robotics, high-throughput instrumentation, computer-aided design, and informatics. The system is engineered to accelerate iterative biological design-build-test-learn cycles, allowing hypotheses to be tested in larger numbers and the lessons learned to be rapidly applied to new experiments. This is demonstrated by expediting plant genome engineering and the characterization of its effects using single-cell metabolomics. The FAST-PB biofoundry uses an existing robotic laboratory called iBioFAB (Illinois Biological Foundry for Advanced Biomanufacturing), a previously developed, scalable, fully automated platform for guide RNA design, preparation of PCR and Golden Gate reactions, *E. coli* transformation, and plasmid isolation as the basis of the design module of the FAST-PB biofoundry (Figure) (Enghiad et al. 2022).

**Figure.**
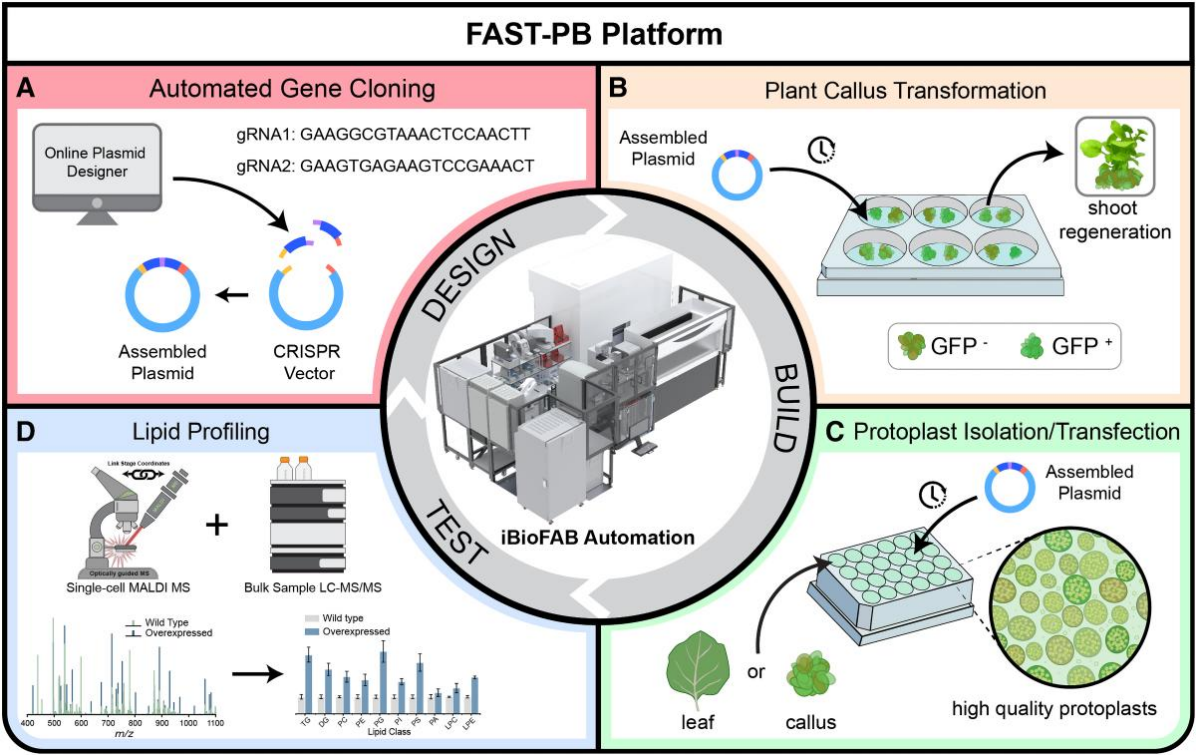
Figure:(**A–D**) FAST-PB is a momentum-based biofoundry with modules such as design, build, and test, designed to study lipid metabolism and engineering. Reprinted from Dong et al. (2025), Figure 1.

Plant protoplast isolation and transformation or transfection is traditionally a time-consuming and labor-intensive process. To address this, the authors first developed and optimized an automated protoplast isolation method that yields high-quality viable protoplasts (Figure). These automated modules were incorporated into the build module of FAST-PB, enabling efficient transformation or transfection of constructs generated in the design module into *Nicotiana benthamiana* or maize (*Zea mays*) protoplast cells. To demonstrate genome editing, the authors also developed a reporter gene–free cellular assay utilizing a single gene, the *High Chlorophyll Florence 136* (*HCF136*), and in the absence of this gene, cells showed higher chlorophyll fluorescence. This allowed them to track genome editing events without the need for traditional reporter genes.

With these advanced tools and automated methods, the authors studied lipid metabolism and engineering, given the crucial roles of lipids in signaling and energy storage and the interest in plant lipids as sustainable fuels. They studied orthologs of genes such as *WRI1* (*WRINKLED1*), *DGAT1* (*Diacylglycerol O-acyltransferase 1*), *Oleosin*, *Thio14* (*thioesterase*), and *LPATB* (*lysophosphatidic acid acyltransferase*) in *N. benthamiana* and/or maize using strong promoter systems and CRISPR activation (Zhai et al. 2017; Vanhercke et al. 2014). The authors observed an increase in the accumulation of various lipid classes following CRISPR activation of selected genes, with a slight further increase when using strong promoter transgene systems, and validated both methods in *N. benthamiana* and maize protoplasts. The authors then expanded the method from protoplasts to callus systems.

In the final module, the authors established a high-throughput, automated method for analyzing lipids at the single-cell (protoplast) resolution, utilizing an integrated matrix-assisted laser desorption/ionization Fourier transform ion cyclotron resonance mass spectrometry (MALDI FT-ICR MS) approach. The lipid profiles derived from individual protoplasts through MALDI FT-ICR MS exhibited a strong correlation with those obtained from bulk callus extracts analyzed via conventional LC-MS/MS lipid profiling techniques. Using the FAST-PB pipeline, the authors also demonstrated successful whole-plant regeneration with lipid profiles in leaf or seed of engineered plants being consistent to those observed in protoplasts or callus. This shows that for some types of experiments, such as lipid engineering, protoplasts can be used for rapid prototyping of different engineering strategies.

Despite these advances, further work is required to automate the entire process of plant regeneration from callus or protoplast without human intervention. For example, FAST-PB still relies for now on manual transfer of calli to shoot media to regenerate transgenic plants. In summary, the development of FAST-PB represents a significant advancement in plant bioengineering. Initiatives like the Global Biofoundry Alliance (https://www.biofoundries.org/) can help accelerate the development of microbial, mammalian, and in vitro systems and extend these capabilities to plant bioengineering.

## Dive Curated Terms

The following phenotypic, genotypic, and functional terms are of significance to the work described in this paper:

Thio14 Gramene: AAC49180.1
Thio14 Araport: AAC49180.1

CpuLPATB Gramene: ALM22873.1
CpuLPATB Araport: ALM22873.1

CpuDGAT1 Gramene: ANN46862.1
CpuDGAT1 Araport: ANN46862.1

SiOle Gramene: Q9XHP2.1
SiOle Araport: Q9XHP2.1
